# Oculomotor Fatigue and Neuropsychological Assessments mirror Multiple Sclerosis Fatigue

**DOI:** 10.16910/jemr.13.4.6

**Published:** 2020-09-13

**Authors:** Wolfgang H. Zangemeister, Christof Heesen, Dorit Röhr, Stefan M. Gold

**Affiliations:** University Medical Center Hamburg-Eppendorf, Germany; MPCH – Medical Prevention Center Hamburg, Germany

**Keywords:** Multiple Sklerosis, Fatigue, Eye Movements, Saccade velocity, Neuropsychological assessment

## Abstract

Fatigue is a major complaint in MS. Up to now no objective assessment tools have been established which hampers any treatment approach. Previous work has indicated an association of fatigue with cognitive measures of attention. Oculomotor tests have been established in healthy individuals as a read-out of fatigue, and to some extent in MS patients. Based on these observations we compared two groups of MS patients, one with fatigue (n=28) and one without fatigue (n=21) and a group of healthy subjects (n=15) with a standardised computerised measure of alertness and an oculomotor stress test. Patients with fatigue showed highly significant changes of their saccade dynamics as defined by the Main Sequence and Phase Plane plots: They showed slowing of saccades, the characteristical fatigue double peak, and an asymmetrical phase plane. Oculomotor tests differentiated significantly between fatigue and fatigabiliy in our MS patients. They also showed significantly worse performance in the alertness test as well as in the oculomotor task. Significantly slower reaction times were observed for tonic alertness in 2 series without a cue (p=.025 and p=.037) but not in phasic alertness with a cue (p=.24 and p=.34). Performance was influenced by disability as well as by affective state. We conclude, when controlling for disability and depression, saccadic stress tests and alertness tests could be used as an objective read-out for fatigability and fatigue in MS patients.

## Introduction

Alertness is the state of active attention by high sensory awareness, such as being watchful and prompt to meet danger or emergency, or being quick to perceive and act. Many studies of vigilance research during the past few decades have shown that, for most or all operators engaged in sustained, attention-intensive and monotonous tasks, retaining a constant level of alertness is rare if not impossible --- leading to alertness deficits and fatigue (94). Posner and Petersen (76) reviewed the breadth of the knowledge about attention in 1990 in his comprehensive examination of the nature and functions of attention and its relationship to broader cognitive processes. An abnormal perception of effort is a salient feature of chronic fatigue. Chronic fatigue is prevalent in a large number of pathological conditions: neurological, autonomic, immunological, hormonal and cardiovascular diseases, and also as a signiﬁcant side effect of many pharmacological interventions. Although the implications of mental fatigue are well known, measuring mental fatigue—especially in complex, dynamic tasks—is still challenging (83). Recent studies investigated mental fatigue using behavioural measures, such as eye movements (79), psychophysics, fMRI and EEG.

Fatigue is a major complaint of MS patients affecting activity and participation substantially. Up to 80% of patients report fatigue and half of those describe fatigue as the worst symptom (Fatigue MS Council 1998). Data indicate that fatigue leads to more unemployment than mobility restrictions (54). During the disease course fatigue persists and may deteriorate (55). Fatigue may be classified into primary and secondary fatigue with the latter being a consequence of disability and reduced activity, sleep disorders, psychological factors, drugs and other medical diseases (58, 14). Factors discussed in the development of primary fatigue are functional changes in the brain such as abnormal recruitment of cortical and subcortical networks in MS (40), fatigue associated with the disruption of frontal and parietal pathways (81), nerve conduction alterations (64), immune and neuroendocrine factors (60), and alterations of muscle metabolism (review in: 58). While there seems to be an association of overall lesion load on MRI and fatigue (23), studies searching for a pattern of lesion distribution with conventional and nonconventional MRI methods did not lead to conclusive results (5, 6). Worsening of fatigue within the first 2 years of MS (17, 92) was a predictor of brain atrophy 6 years later: Yaldizli et al. (100) have shown correlations of fatigue scores with corpus callosum atrophy in 70 relapsing-remitting MS patients.

Any study on MS fatigue is hampered by the fact that up to now no objective assessment methods have been established. Monitoring of its natural course as well as under experimental treatments necessarily relies on self-report questionnaires. A study in unselected RRMS patients indicated that longer reaction times in a computerized test of alertness, i.e. sustained attention may be associated with MS fatigue (96). Maclachlan et al. (66) examined whether current chronic fatigue syndrome (CSF) diagnostic criteria are identifying different disease phenotypes using the DSQ-DePaul Symptom Questionnaire. Self-reported autonomic and cognitive symptoms were significantly greater in CFS subjects compared to controls (93). There were no statistically significant differences in objective autonomic measures between CFS and controls.

Based on the concept that the ascending arousal system and the Locus coeruleus are major determinants of wakefulness and attention (51) the oculomotor system, located in close proximity to these systems, might be an objective read-out for disturbed brain stem functioning and may therefore serve as a substrate of MS fatigue (9, 13, 65, 10). Indeed, reduced velocity of horizontal saccades has been associated with tiredness in sleep deprived healthy individuals (106, 79). Recently, Serra et al. (82) have reviewed the most common eye movement disorders in MS and discussed known pathophysiological correlates for each of them. Study of eye movements in MS is particularly valuable as use of conventional and upcoming non-conventional imaging techniques coupled with eye movement recordings can provide particular insights into the course of the disease.

Fatigue can be subdivided into an effort-independent (trait-fatigue) (50) and an effort-dependent component (fatigability) (57). Spiteri et al. (88) tried to disentangle activity changes associated with effort-independent “trait-fatigue” from those associated with effort-dependent fatigability in MS patients through behavioral measures and functional magnetic imaging. Their results indicated that effort-independent (fatigue) and effort-dependent fatigue (fatigability) in MS patients have functionally related but fundamentally different neural correlates. Saccadic velocity is not subject to voluntary control (63), unlike saccadic amplitude or fixation duration. Thus it represents the underlying neural activity more accurately than other gaze parameters (15, 38). Saccadic eye movements and their neurological control signals change significantly as the human subject fatigues. Dodge and Cline (33) were probably the first to describe the dynamics of saccades and changes of their dynamic due to fatigue with decrease velocities (32), and later Bahill and Stark (3). With increased attention saccade dynamics change to the opposite and very fast saccades can be found indeed (42). A different new method for the analysis of eye movements and scanpaths has been proposed by Hein and Zangemeister (52). They applied the theory of topology for addressing the relationship between spatial features of eye movements and their visual-objects.

Purpose of this study. Based on the above noted earlier observations our study addressed the question whether using an objective, computerized measure of alertness and a horizontal saccadic stress task would allow to differentiate MS patients with and without fatigue and/or fatigability. We compared two groups of MS patients, one with fatigue, one without fatigue and a group of healthy subjects with a standardised computerised measure of alertness and an oculomotor stress test. Our hypothesis was that when controlling for disability and depression, saccadic stress tests and alertness tests could be used as an objective read-out for fatigability and fatigue in MS patients.

## Methods

Recruitment and inclusion/exclusion criteria Patients with clinically definite MS according to McDonald criteria (68), age 18-60, Expanded-Disability-Status-Scale Scores (**EDSS**, 62) 0-6, and the ability to read were allowed to participate. The database of the MS Outpatient clinic in Hamburg was screened for patients with substantial fatigue based on their responses on the fatigue items of the Hamburg Quality of Life Questionnaire for MS **(HAQUAMS)** (46). Patients had to be untreated or on stable disease-modifying therapy for at least 3 months. Furthermore, patients were excluded if they had received steroid treatment within the last 4 weeks. For demographic and clinical characteristics of patient sample see Tab.1 (78).

### Fatigue scoring

Patients were grouped into Fatigue and Non-Fatigue based on the score on the Fatigue Severity Scale **(FSS,** 61, 60) according to a published cut-off (FSS score >4). In addition, patients completed the Modified Fatigue Impact Scale (**MFIS,** MS Council 1998) and the Beck depression inventory (**BDI**, 11).

### Clinical measures

Neurological impairment was assessed using the **EDSS (**Expanded Disability Status Scale**)**. In addition, we obtained clinical disease course and disease duration. Cognitive function was screened with the Symbol-Digit-Modalities-Test (**SDMT**, Symbol Digit Modalities Test, 85), a sensitive screening measure. Hand function was controlled by use of the **9-HPT** **(**Nine*-*Hole-Peg-Test*,* 26).

### Eye movement recordings

All subjects had normal or corrected-to-normal vision. Only non-smokers were recruited and they had to abstain from alcohol 24 h and from caffeine-based drinks 12 h before participating. All participants slept at least 7 h before being evaluated. In order to avoid confounding influence of circadian rhythm (98) or any diurnal variation (48), all experimental sessions were conducted between noon and 4p.m. during the day. The study was conducted in conformity with the declaration of Helsinki (WMA, 2008).

### Infrared - Oculogram (IR-OG) - Instruments.

We used a 22-inch monitor (74Hz) of 1.4 cd/m^2^ background luminance. The horizontal visual angle of the stimulus (a white cross of 1 deg diameter and 20.6 cd/m^2^ luminance) was ± 5, 10, and 15 degree. To prevent head movements, a head fixation device that tightly strapped the head with a circular head holder was attached to the forehead. Participants were seated comfortably with a viewing distance of 57 cm (1° is equivalent to 1cm). Under standardized conditions, the eye movements were recorded with infrared reflection oculography (OBER JazzNovo). We used a high resolution software (Eye Track Project 1.21: 102); 101, 72) to present horizontal stimuli and pre-analyze the recordings using Main Sequence characteristics of recorded eye movements (2, 4) that were used for later statistical comparisons (duration, peak velocity, peak positive acceleration). Eye blinks, which appeared in the original data were removed by computer editing. Sampling time was at 1000 Hz using the Ober JAZZ NOVO remote eye tracking system with an average spatial accuracy of <0.1°. Saccades and fixations were measured using the saccade detection algorithm supplied by JazzNovoResearch (see Appendix). Saccades were identified by deflections in eye position in excess of 0.1°, with a minimum velocity of 30°/s and a minimum acceleration of 4000°/s^2^, maintained for at least 4 ms. A nine-point calibration and validation was performed before the start and at the end of each session. Saccades around blinks, as well as fixations shorter than 100ms, and saccades with durations less than 10 ms, were not considered in the analysis.

### Experimental protocol, stimuli, stress test

Prior to each session and block, calibration and accuracy were tested. Calibration was repeated up to three times prior to each session. Each subject was measured during a standardized routine that consisted of

(1) calibration,

(2) for 10 sec each sinusoidal pursuit eye movement of 0.7 Hz, predictive square wave jerks of ± 10 deg, and random square wave jerks between 2deg and 30deg.

(3) To induce oculomotor fatigue in our 3 groups, participants had to complete four optokinetic (30°/sec) stress tests of 4 minutes each: alternating between right and left direction.

(4) for 10 sec each sinusoidal pursuit EM of 0.7 Hz, predictive square wave jerks of ± 10 deg, and random square wave jerks between 2deg and 30deg. Total test duration was about 20 minutes.

The recorded single values were transferred into a table that showed: Latency, Duration, Amplitude, peak velocity, peak acceleration and deceleration, times of these maximum values, and averages of these comparing left- and rightward saccades. These values were displayed by means of the double logarithmic Main Sequence graph (2) comparing them with normal values of our lab (102, 72). See appendix for velocity and acceleration analysis formulas.

### Phase plane analysis of saccades

When two time trajectories are plotted against each other, phase-plane plots are formed. Phase-plane plots of dependent variables and their derivatives are often used to display behavior without explicit time dependency. With respect to the dynamics of saccades, Cook et al., (22), and Clarke and Stark (21) where the first to apply this tool. Later, Zangemeister and Stark (01), Peng et al. (74), and Benko et al. (12) also used it for description of head-eye movement dynamics with respect to the active VOR (vestibular ocular reflex).

**Figure 1.**
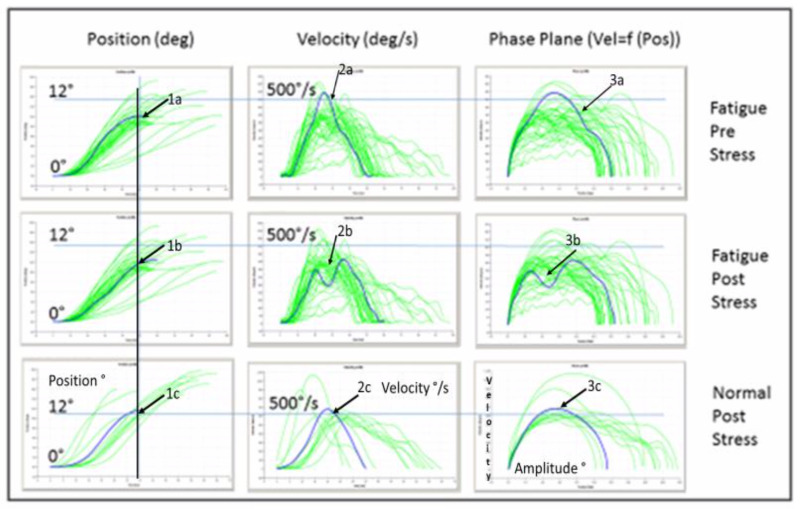
Characteristics of amplitude, velocity, velocity/amplitude phase-plane of MS fatigue patient SC. Before [pre] and after [post] stress test (upper two rows). Median curves of 30 samples. For comparison, lowermost row shows corresponding graphs of a healthy subject (KF) after stress test, with normal dynamic and phase-plane graphs, even after the stress test*.* Note the saccadic amplitude reduction (arrow 1b) and significant reduction of peak velocity (2b) after the stress test, compared to before stress test (1a and 2a); falling below the two sigma main sequence limits of healthy subjects aged 20 to 60 in our lab. Note also the typical dynamical fatigue effects of the patient´s saccades with double velocity peaks (2b) [already slightly indicated before stress test (2a)]. Double saccades (3b) and asymmetrical (3a) phase-plane graphs demonstrate this result as well. Axes. Ordinates: Left: position, deg. Middle and right: velocity, deg/s. Abszissae: Time, 0 to 100 ms. Left vertical line depicts time when final saccadic amplitude has been reached: after 35ms (lowermost: normal), uppermost and middle (fatigue) after 50ms and 60ms respectively.

**Figure 2.**
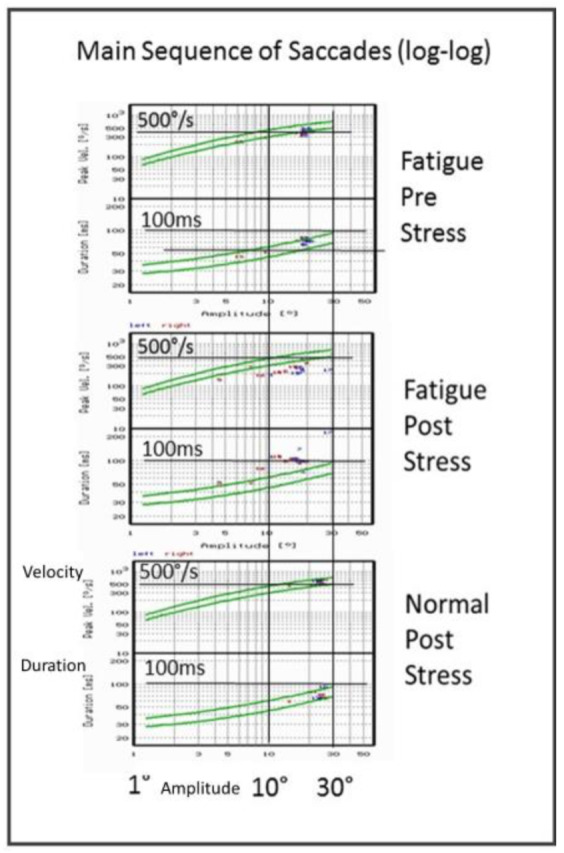
Characteristics of Main Sequence (log-log) graphs of MS fatigue patient SC before [pre] and after [post] stress test (upper two graphs), and a normal healthy subject for comparison (lowest graph) [post] stress test. Each panel shows peak velocity in deg/s upper, and duration (sec) lower as functions of amplitude (deg). Note the saccadic amplitude reduction and significant reduction of peak velocity after the stress test; falling below the two sigma main sequence limits of healthy subjects aged 20 to 60 in our lab.

These plots provided the clearest and most efficient means for comparing single time trajectories. Large amounts of data from multiple saccades could be presented in a compressed format using the phase-plane plots. The time trajectories of the eye in amplitude and velocity, and the corresponding phase plane plots during multiple saccades before and after the stress test of a MS fatigue patient are presented in Fig. 1. The two uppermost rows, display a response from a MS fatigue patient (during multiple predictive saccades of +-10°). We created a phase plane in which the saccadic velocity trajectories are plotted against the saccadic amplitude trajectories (thick solid lines in the time trajectories shown in the top panels). Fig. 1, lowermost row displays a response from a healthy subject *after the stress test,* since there was no difference between pre and post stress test; the corresponding phase planes of the time trajectories are shown on right. The log-log Main Sequence graphs are shown in Fig.2. For outcome measures derived from the IR-OG, normality of data distribution was tested using the Kolmogorov-Smirnov Z test. Results indicated no violation of the normal distribution assumption for these parameters. Thus the two tailed students t-test was applied.

### TAP – alertness

Alertness refers to the condition of general wakefulness that enables a person to respond quickly and appropriately to any given demand. It is the prerequisite for effective behavior, and is in this respect the basis of every sustained attention performance. This attention test has a low complexity with simple reaction paradigms. In the subtest alertness of the *Tests for Attention **(*****TAP**) (107), reaction time is examined under two conditions. The Test consists of two components, which can be divided into four series with 75-90 sec duration each. A key is pressed when a crosshair (easily discriminated speech-free stimulus) appears in the middle of the screen. The reaction speed is recorded in "ms". The crosshairs disappear when a key on the PC keyboard is pressed. After an unpredictable time period a cross appears again. Each series contains 20 individual evaluations (56).

The testing was carried out according to the ABBA scheme. In the series 1 and 4 (A) the determination of the general reactivity (Tonic Alertness) is done by the appearance of the crosshair on the image surface. In series 2 and 3 (B) the phasic alertness is tested. Here the subject is required to perform a more complex, dual-task. At the same time, two stimulus presentations must be observed, which include the visual task and an acoustic task. In the visual task, the respondent must register a cross appearing on the monitor and in the acoustic task she must practice the perception of a high-pitched tone followed by the appearance of the crosshairs in temporal independence of the signal tone.

### Statistical analysis

Clinical and demographic variables between the groups were compared using independent samples t-tests or chi-square tests as appropriate. For outcome measures derived from the TAP, normality of data distribution was tested using the Kolmogorov-Smirnov Z test. Results indicated violation of the normal distribution assumption for most TAP coefficients. Thus, these group comparisons were tested using non-parametric Mann-Whitney U tests for independent samples. Statistical analyses were performed using IBM/ PASW software.

### Test-abbriviation

**TAP** *Test battery for attention*

**MFIS** *Modified Fatigue* I*mpact Scale*

**MFIS** **Cog** *mfis cognition*

**MFIS Physe** *mfis* *physical*

**MFIS PsySoz** *mfis psychsocial*

**SDMT** *Symbol Digit Modalities Test*

**EDSS** *expanded disability status score*

**9-HPT** *Nine-Hole-Peg-Test*

**PASAT** *paced-serial addition-task*

**MACFIMS** *validity of minimal assessment of cognitive function in MS*

**BDI** *Beck-Depression-Inventory*

**FSS** *Kurtzke Functional Systems Scores*

**RRMS** *Relapsing Remitting Multiple Sclerosis*

**HAQUAMS** *Hamburg Quality of Life Questionnaire for MS*

## Results

### Demographic and clinical characteristics

A total of 49 MS patients were included in the study. According to the predefined cut-off (FSS>4), 28 patients were classified as fatigue, and 21 were classified as non fatigue patients. There were no significant differences in age, disease duration, or disease course between the fatigue and the non-fatigue group (Tab.1). Fatigue patients had marginally higher EDSS scores (p=.06); the fatigue group included a slightly higher percentage of female patients.

Using the ratio of saccadic peakVelocity/ Amplitude, which is the important indicator of the Main Sequence as described by Bahill et al. (2) and its variance by Bahill et al. (4), we used the single straight forward parameter to define dynamics of fatigued saccades.

**Table 1.** Demographic and clinical characteristics of patient sample.

	MS Fatigue (n=28)	MS non-Fatigue (n=21)	P*
Age	41.7 +-1.4	40.05+-2.16	n.s.
Gender (F/M)	26 / 2	15 / 6	0.06
Disease duration	6.00+-1.0	5.62+-1.1	n.s.
EDSS	3.0+-0.1	2.3+-0.3	0.06
SDMT	56.4+-1.6	60.1+-3.1	n.s.
9-HPT	18.2+-0.5	18.2+-0.7	n.s.

Note. *according to independent samples t-tests

As expected, significant group differences were observed in fatigue scores for cognitive, physical and psychological fatigue symptoms (Table 2). In addition, fatigue patients showed higher levels of depression as measured by the BDI.

**Table 2.** Self-report measures of fatigue and depression.

	MS Fatigue (n=28)	MS non-Fatigue (n=21)	P*
FSS	5.60+-0.15	1.73 +-0.24	p< .001
MFIS phys	23.25+-1.18	6.00+-1.28	p< .001
MFIS cog	25.68+-1.30	6.95+-1.68	p< .001
MFIS psych	4.39+-0.35	0.86+-0.27	p< .001
BDI	14.17+-1.25	4.86+-0.96	p< .001

Note. *according to independent samples t-tests

Using the ratio of *peakVelocity/ Amplitude*, both of which are the main indicators of the Main Sequence as described by Bahill et al. (2), we found highly significant differences between the groups: Before and - more enhanced -, after the stress test. It is crucial to reduce the variability of the quantification of saccadic dynamics by i. defining accurately the saccadic amplitude and ii. the peak velocity that belongs to the found amplitude – falling within or outside the normal Main Sequence. In case of non-fatigue compared to fatigue MS patients a greater difference after the stress test would be expected.

The normal healthy group for comparison showed normal values of all parameters pre and post stress as well, (student´s t-test in confirmed Gaussian distribution of IR-OG values). The stress test had no effect in the non-fatigue MS patients, but a significant effect in the fatigue group. The initial -pre stress test- comparison of the non-fatigue with fatigue group did not show a difference; but after the test we found a significant difference within the fatigue group. The initial pre- stress test values of the fatigue group showed a highly significant difference to those after the test. This showed that the fatigue patients´ initial condition was significantly worse than in the non-fatigue group. Using in addition the stress test reveals an even more pronounced difference. Using both discriminatory values gave us a clear and reliable objective measure in our cohort of fatigue MS patients.

**Table 3.** Statistical results (student´s t-test in confirmed Gaussian distribution of IR-OG values) of infrared oculography in MS patients with and without fatigue before (pre-) and after (post-) the oculomotor stress test.

pV/Ampl	N	Pre stress test P*	Post stress test P*
NOR	15		0.42
NOR/ MS-Non-Fatigue		0.29	*0.05*
MS Non-Fatigue	21		0.1
MS Non-Fatigue/ MS Fatigue		0.83	*0.01*
MS Fatigue	28		*0.0005*
NOR/ MS Fatigue		0.24	*0.0001*

Shown in Fig.1 are median curves of 30 samples before [pre] and after [post] stress test (upper two rows). They demonstrate the saccadic amplitude reduction (arrows 1a&1b), and also the significant reduction of peak velocity after the stress test (arrow 2b), compared to before the test (arrow 2a). Saccades with double velocity peaks (arrow 2b) demonstrate the dynamical fatigue effect on saccade dynamics. These are double saccades as seen also in asymmetrical (3a) and double (3b) phase-plane graphs. They lie below the two sigma main sequence limits of healthy subjects aged 20 to 60 as shown in figure 2.

The left vertical line in Fig.1 depicts the time when final saccadic amplitude has been reached: normally this is after 35ms (lowermost), but only after 60ms and 50ms respectively in case of stress-induced additional fatigue in the patient (middle and uppermost).

In comparison, post stress saccade dynamics of the heatlthy subject show correct amplitude (1c), same velocity as pre test (2c), and symmetrica , non-double peaked phase planes (3c).

Main Sequence (log-log) graphs of MS fatigue patient SC (Fig.2). The results depicted in Fig.1 show up also in the double log Main Sequence graphs of Fig.2. Before stress test (uppermost) already slow peak velocity values are seen in the Main Sequence of a patient. A significantly increased effect shows up after the stress test (middle) with lowered peak velocity values.

Similarly, normal duration values are seen in the fatigue patient before (uppermost) the stress test that increase significantly (middle) after the stress test.

In the healthy subject (lowermost) normal saccade dynamics are seen, even after the stress test for the peak velocity values. The same is true for the duration values.

### Influence of disability and affective state

During the TAP alertness paradigm we observed significantly longer reaction times in patients with fatigue (Table 4). Significantly slower reaction times were observed for tonic alertness (series 1 and 4), but not for phasic alertness (series 2 and 3).

**Table 4.** TAP alertness in MS patients with and without fatigue

	MS Fatigue (n=28)	MS non-Fatigue (n=21)	P*
Series 1	397.3+-44.9	260.7+-13.9	.025
Series 2	352.3-37.2	268.6+-11.5	.24
Series 3	348.0+-38.3	261.6+-10.8	.31
Series 4	412.6+-53.4	282.6+-17.8	.037

Note. *according to Man Whitney U test

Since there were slight differences in EDSS and gender and significant differences in depression scores (BDI) between the fatigued and the non-fatigued patients, we next explored the associations between these variables and the TAP results in order to rule out potentially confounding effects. No significant differences were found in the TAP performance between female and male patients (all values p >.20), indicating that differences in gender distribution are unlikely to confound the reported results in TAP scores. But there were significant correlations between EDSS and TAP reaction times (Spearman Rank coefficients Tab.5, second row). After statistically controlling for EDSS, group differences between fatigued and non-fatigued MS patients remained significant for TAP series 1 (p=.032) but not TAP series 4 (p=.113).

BDI total score significantly correlated with some of the TAP reaction times (Spearm.Rho: series1 r=.41,p=.004; series4 r=.34, p=.02). Since the BDI contains numerous items covering vegetative symptoms of depression, which overlap with symptoms of fatigue, we next examined the impact of depression after removing those items. The affective subscore of BDI adressing six purely depressive symptoms (items A, B, E, F, I , J) showed no significant associations with TAP reaction times (Tab.5 third row). This indicates that pure affective symptomatology is unlikely to confound group differences in TAP scores between fatigued and non-fatigued MS patients.

**Table 5.** Correlations ( Spearman Rho) TAP and SDMT, EDSS, BDIaff, 9-HPT.

Parameter	TAP Series 1	TAP Series 2	TAP Series 3	TAP Series 4
FSS	0.389**	0.220*	0. 177	0.351*
EDSS	0.33*	0.29*	0.29*	0.36*
BDI affect	0. 185	0. 55	0. 092	0. 127
9-HPT	0.375*	0.418**	0.466**	0.380*

Note. *significance <0.05, ** significance <0.01

Both FSS and nine-HPT showed high correlations with the TAP test.

**Table 6.** Correlations (Spearman Rho) between IR-OG results after stress test with MFIS Cog, 9-HPT.

	MFIS		9-HPT	
Saccade	MfisScore Rho	P value	9-HptScore Rho	P value
Peak + Acceleration	0.307	0.004	-0.307	0.01
Peak Velocity	0. 300	0. 01	-0.321	0. 02
Amplitude	0.265	0.09	0.377	0.05
Duration	-0.311	0.05	0.103	0.110

Table 6 depicts the correlations between MFIS Cog and Nine-HPT with the oculomotor parameters after the stress test. The MFIS Cog (10 items) is a modified subtest version of the Fatigue Impact Scale, designed as a self- report measure to rate fatigue in MS. As expected correlation with saccade dynamics is very high, particularly for peak positive acceleration. This is also true for the Nine HPT as a motor performance test. The table demonstrates that correlation of the dynamic parameters i.e. peak positive acceleration and peak positive velocity with both neuropsychological tests is highly significant, compared to amplitude and duration that are less valuable in this respect.

## Discussion

This study assessed two functional brainstem tests, saccade dynamics and a measure of alertness as possible correlates of MS fatigue. Based on earlier observations we compared two groups of MS patients, one with fatigue and one without fatigue with a group of healthy subjects using standardised computerised measure of alertness and an oculomotor stress test. Patients with fatigue showed highly significant changes of their saccade dynamics as defined by the Main Sequence and Phase Plane plots. They showed slowing of saccades, the characteristical fatigue double peak, and an asymmetrical phase plane. The oculomotor tests differentiated significantly between fatigue and fatigabiliy in our MS patients. They also showed a high correlation between the alertness tests and the oculomotor task. Significantly slower reaction times were observed for tonic alertness in two series without a cue, but not in phasic alertness with a cue. Performance was influenced by disability as well as by affective state. In the second part of the discussion section you should compare your results to past studies, particularly studies discussed in the introduction. If the results are not the same, discuss possible reasons for the difference.

The oculomotor system has been a focus of fatigue research. Unlike saccadic amplitude or fixation duration, saccadic velocity is not subject to voluntary control (63), and therefore represents thus it may represent the underlying neural activity more accurately than other gaze parameters (15). Saccadic eye movements and their neurological control signals change significantly as the human fatigues: Dodge (1). High resolution recordings of anomalous looking saccadic eye movements that occurred as the subject's physiological state changed to fatigue were analyzed by Bahill et al. (2, 3). They described the neuromotor control signals for differentiating saccades during fatigue: 1. Overlapping saccades in which the high-frequency saccadic motoneural bursts showed large pauses; 2. glissades in which the high-frequency motoneural bursts were much shorter than appropriate for the size of the intended saccades; 3. and low-velocity, long-duration, non-Main Sequence saccades in which the motoneuronal bursts were of lower frequency and longer duration than normal. As few as 30 saccades of 50 deg magnitude or a longer sequence of small saccades of 10 deg could produce these aberrant eye movements. These effects of fatigue explain most of the variations between published data of velocity to amplitude rate of human saccadic eye movements – the Main Sequence (2). The fatigue noted in these saccades was probably manifested at several levels. In the typical fatigue sequence glissades -slow corrective movements of the eye- were the first signs of fatigue to appear. In these eye movements the control signal pulse duration did not carry the eye as far as normal. This could have been due to fatigue of the extraocular muscles or their motoneurons. Later, in order to compensate, the CNS might have changed its stratagem to produce double saccades or low-velocity, long-duration, non-Main Sequence saccades (3). Dieter Schmidt et al. (80) described these effects in mental and muscular fatigue upon saccade velocity.

### Decrease of peak saccadic velocity

Injections of diazepam, which increases presynaptic inhibition in the spinal cord, and affects the limbic system and cerebellar structures, produced double saccades and low velocity saccades (1). Also, alcohol produced similar decrements in the speed of saccades (32). Drugs that affect the GABA/benzodiazepine receptor influence also attention and cognitive activity as well as saccade velocity (8). Clonidine led to peak velocity decreases. Grace et al. (48) reported that morphine-induced sedation and sleep deprivation lowered peak velocity. Reduced arousal due to task features (29, 28) and sleepiness (53) lead to decreased saccadic velocity. In Parkinson´s disease attention may be decreased and in conjunction saccades slowed (104, 20, 16, 103, 73).

Already in the early years of eye movement research, decreased saccadic speed had been consistently found in healthy controls during high continuous mental load and sleep deprivation (69). Matta et al. (67) studied fatigability of horizontal saccades in 9 multiple sclerosis patients with clinically proven internuclear ophthalmoplegia (INO). INO patients with mild affection showed worsened conjugacy during the 10 minute fatigue test, while more severe INO patients showed improved conjugacy perhaps due to adapative mechanisms as the authors speculated. However, chronic fatigue patients with INO do not appear to be a good model to give a specific measure of both chronic fatigue and /or fatigability. The Matta et al. (67) article reports on MS patients who suffer already from an INO that is it originates from a non- peripheral, central paresis that is defined by a distinct lesion of the internuclear brainstem connection with the result of slowed nasal saccade(s). In fatigue of MS patients however, we have not a distinct lesion but a more general state of fatigue that can involve all saccadic movements and their dynamics; i.e. not just one particular connection as in INO. Also, this general state involves more than the brainstem connectivity and arousal system, as the bulk of reports about fatigue in MS patients show. So, we feel that a distinct localized brainstem lesion like the INO would interfere with a general state of fatigue: Fatigue signs in saccades then would have to be disentangled from velocity changes due to zentral pareses caused by an INO.

As a fatigue-test our suggested method had the advantage that it records both trait-fatigue (before the stress test) and fatigability (after the stress test). If one would find already at the test start an INO, the general aspect of trait-fatigue could not properly be disentangled. Therefore patients with INO or other special pareses of saccades should be excluded from this test – what we did.

Di Stasi et al. (30) showed that microsaccades and drift dynamics reflect mental fatigue; they showed that saccadic and microsaccadic velocity decreased with time-on-task whereas drift velocity increased, suggesting that ocular instability increases with mental fatigue. Task *difficulty* did not influence eye movements despite affecting reaction times, performance errors and subjective complexity ratings. They concluded that changes in fixational and saccadic dynamics can indicate mental fatigue due to time-on-task, irrespective of task complexity.

### Increase of saccadic peak velocity

Idazoxan, which generally increases arousal, led to saccadic peak velocity increases (24). Also, Increased arousal due to drug use (45), increased motivation (i.e. due to reward) (91), and perhaps increased effort (44, 30) can raise saccadic velocity.

These effects of arousal on saccadic peak velocity could arise at the level of the excitatory burst neurons, whose firing rates encode the velocity signal of saccades (34, 86, 87, 106). Changes in attentional processing --for instance, due to variations in arousal-- can affect the strength of the excitatory connections from the frontal cortex to the brainstem reticular formation (71), thereby changing the characteristics of the main sequence. Arousal may affect peak velocity via the inhibitory connections between the sleep-regulating centers --- nucleus raphe magnus, nucleus raphe dorsalis, and locus coeruleus--- and the superior colliculus on the reticular formation and cerebellum.

### Pupillary oscillations

Pupillary oscillations as an indicator of central autonomic nervous system tone have been associated with tiredness (97). However, in a study comparing MS patients (with minor disability) and healthy volunteers, Egg et al. (35) could not show a correlation of increased pupillary oscillations with fatigue scales (19). Matta et al. (67) has studied horizontal saccades in MS as a model for central fatigability. However, subjectively perceived fatigue was not assessed and all their patients did show an internuclear ophthalmoplegia, INO, which our patients did not. Therefore, central nerve conduction changes or subclinical pareses might have accounted for the findings in their study.

### Level of fatigue symptoms

Our results confirmed our prediction that the level of fatigue symptoms significantly reduced the characteristic Main Sequence ratio of peak velocity to amplitude due to saccadic fatigue as described by Bahill et al. (2, 3). Neurophysiological evidence indicates that ocular motor fatigue is of premotor and brainstem origin, rather than of muscular origin. These premotor processes likely reflect altered cortical or cerebellar influences that might result in a decreasing alertness, i.e. the ability to sustain attention (77, 7, 90). The functional paradigm used in our study is likely to capture pre-motor changes in the oculomotor system (see also 41) and requires top-down control of the eye movements (39, 95).

### Variability of peak velocity

As seen in Table 3, slowed peak velocities in MS patients with fatigue were also found in patients with non-fatigue in our cohort. This points to the variability of saccade dynamics particularly when we do not look at the lead parameter of the main sequence which is the relation between amplitude and peak velocity, i.e. the ratio of these two. Defining saccades through their exact amplitudes permitted us to link the correct peak velocity to each saccadic amplitude. with low variability of the pV/Amplitude ratio (4). Mental fatigue. For the clinician, the difference before and after the stress test is of importance. Our findings explain the divergence and variability of reports about saccadic dynamics of MS patients with and without fatigue. In our view only by using a stress test similar to the one that we have described, a useful result could be obtained; i.e. a significant and reproducible difference between saccade amplitude velocity relationship and fatigue versus non-fatigue MS patients.

In monkeys, Straube et al. (89) described that after 2000 to 7000 saccades in the dark, peak eye velocity on the average decreased by 20%, and showed increased variability. In contrast, when testing was done in dim light, there was little to no change in average saccadic metrics and latency. These changes in saccadic metrics and dynamics in the dark did not reflect a change of the ocular plant but reflected a change in the cortical or cerebellar influences on the brain stem burst generator linked to the monkeys' attentional state. They showed that this slowing of saccades depended not on fatigue of the extraocular muscles but on mental fatigue and concluded that saccades provide an objective measure of mental fatigue which central processes mediating saccades are responsible for.

### Fatigue and deficits of alertness in sustained attention

An association between fatigue and deficits of alertness/sustained attention has been described by Flachenecker and Meissner (43). They reported cases of severely impaired sustained attention performance, especially in the alertness subdomain in the TAP during a MS relapse perceived as severely increased fatigue which resolved after treatment. They concluded that measures of alertness, i.e. sustained attention might be objective correlates of MS fatigue. Di Stasi et al. (29, 30) showed that slowing of saccadic velocity is a reliable indicator of the subjective fatigue of health care and other professionals during prolonged time-on-duty. Time-on-duty decreased saccadic velocity and increased subjective fatigue but did not affect surgical performance. These results supported the hypothesis that saccadic indices reflect graded changes in fatigue.

### Chronotype influence

Cazzoli et al. (18) demonstrated that mean saccadic speed significantly decreased throughout the duration of a fatiguing task, but was not influenced by the optimal or non-optimal time of the day for both chronotypes, i.e. a ‘morning type’ or an ‘evening type’. The results suggested that different oculomotor parameters are discriminative for fatigue due to different sources. A decrease in saccadic speed may have reflected fatigue due to time spent on task. An increase in mean fixation duration may have shown a lack of synchronicity between chronotype and time of the day.

Many neurocognitive correlates of subjectively perceived fatigue have been studied in the past. The largest cross-sectional and longitudinal cohort compared fatigue scores and the MACFIMS cognitive battery in an unselected sample of n=465 patients (70, 105). No specific measures of alertness were applied. In this study, only the SDMT showed differences between MS patients with fatigue and without fatigue. Walker et al. (95) postulated a correlation of PASAT performance and subjectively perceived fatigue measured by the Fatigue Impact Scale but showed only modest correlations of up to 0.30.

### Fatigue and Depression

Weinges-Evers et al. (96) were the first to show FSS scores as predictors of alertness performance although with a low standardized beta coeffcient of 0.298 in a cohort not selected based on fatigue complaints. However, their study also showed substantial correlations of fatigue scores with disability and depression ratings. While in our cohort depression was more prevalent both cohorts showed that depression and disability impact on fatigue ratings. Melancholic and non-melancholic depression (84) are subtypes of major depressive disorder, each having distinct cognitive and motor impairments (99). They found abnormal main sequences in patients with melancholic depression (decreased saccadic velocity), and relatively normal main sequences in patients with non-melancholic depression. We demonstrated that performance in the attention test was different in MS patients with fatigue compared to a control cohort without fatigue; MS patients with fatigue showed impaired tonic alertness in the applied tests. However, disability and depressive symptomatology also impacted on TAP results, underlining that this objective functional test only reflects partial effects of MS fatigue.

The correlation of fatigue with depression and disability has repeatedly been studied (36; for review see 14). Already in 1908 Diefendorf and Dodge (27) studied saccadic velocities in mentally ill patients, anticipating that saccadic metrics might help to diagnose psychiatric disease. They found abnormal unusually fast saccades in manic patients, but slow saccades in extremely depressed patients. More recently Gooding and Basso (47) performed saccadic research in psychiatric populations. E.g. in normal healthy subjects, execution of visually guided saccades improved the accuracy of corrective saccades made after the first memory-guided saccades to drive the eyes closer to the target, and this improvement was independent from the number of the visually guided saccades Colnaghi et al. (25). In patients with depression this capability is significantly reduced. Visually guided saccades therefore provide a template that improves the capability of corrective saccades to compensate for the residual position error at the end of the first saccade to the memorized goal.

Due to the overlap between fatigue and vegetative symptoms of depression, it is difficult to dissociate these two closely linked symptom domains (75). However, through screening of large cohorts of patients one might be able to define a subgroup with high levels of fatigue but very low levels of depression. On the other hand such an approach by definition reduces the generalizability of the findings for MS-associated fatigue.

## Conclusion

As fatigue is always a self-reported symptom, while depression can be identiﬁed and diagnosed by an external observer, i.e. an objective sign, it suggests that one part of fatigue is a perceptual, inference chronic phenomenon (59). Our measures assessed facets of perceived fatigue that are substantially influenced by disability and affective state. On the other hand, we demonstrate the central neuro-motor part of fatigue which can be objectively measured, as the sense of the oculomotor activity is different from general skeletal muscular sense. When controlling for disability and depression, saccadic stress tests and alertness tests could be used as an objective read-out for both fatigability and chronic fatigue in MS patients. Oculomotor tests differentiated significantly between fatigue and fatigabiliy in our MS patients. Patients with fatigue showed highly significant changes of their saccade dynamics as defined by the Main Sequence and Phase Plane plots. They showed slowing of saccades, the characteristical fatigue double peak, and an asymmetrical phase plane. Further work should study these outcomes in longitudinal studies as well in clinical trials of pharmacological or behavioral interventions.

### Ethics and Conflict of Interest

The author(s) declare(s) that the contents of the article are in agreement with the ethics described in http://biblio.unibe.ch/portale/elibrary/BOP/jemr/ethics.html and that there is no conflict of interest regarding the publication of this paper.

## Appendix

### Appendix 1

Jazz Manager (version 3.13) uses the following

eye velocity calculation formula:

Ev[n] = ( 300 Ep[n-5] - 294 Ep[n-4] - 532 Ep[n-3] - 503 Ep[n-2] +296Ep[n-1] + 296Ep[n+1] +503Ep[n+2] + 523 Ep[n+3] + 294 Ep[n+4] - 300 Ep[n+5]) / (5140 * h)

where: Ev[n] - eye velocity sample at moment n. Ep[n] - eye position sample at moment n. And h - sampling interval (0.001 s for 1kHz sampling).

the eye acceleration calculation formula is:

Ea[n] = ( 2 Ep[n-3] - 27 Ep[n-2] +270Ep[n-1] - 490 Ep[n] + 270Ep[n+1] - 27 Ep[n+2] + 2 Ep[n+3] ) / (180 * h^2)

where: Ea[n] - eye acceleration sample at moment n. Ep[n] - eye position sample at moment n. And h - sampling interval (0.001 s for 1kHz sampling).

**Inside IR-OG data**

The standard deviations of our IR-OG data were small because we plotted the parameters as functions of the size of the actual saccade, not as functions of the size of the target movement and the software calculated the parameters from the velocity trace, derived with a zero-phase digital filter. We defined the size of the saccade as the size of the initial, dynamic saccade. Eye movements with long glissades or drifts were discarded and the magnitude of the dynamic saccade was carefully calculated. For saccades with overshoot, the saccadic magnitude was defined to be the foot-to-peak angle, i.e., the angle between zero velocity at the start of the saccade and zero velocity at the end of the saccade. Less variability was provided by this definition for the size of the saccade than when the size of the saccade was defined as the angle between the two steady state fixation points. Saccades with dynamic overshoot were treated as two saccades: the main saccade and a small return saccade. We then used the ratio of peakVelocity/ Amplitude, which is the main indicator of the Main Sequence as described by Bahill et al. (1975a), for specifically analyzing and comparing the the group data. This permitted to narrow the otherwise relatively high variability of the single dynamic saccade parameters (see Bahill et al. 1981 for an overview of the causes of dynamic variance of saccades).

**The standard deviations of our data were small**

The standard deviations of our data were small, because we plotted the parameters as functions of the size of the actual saccade, not as functions of the size of the target movement and the software calculated the parameters from the velocity trace, derived with a zero-phase digital filter. We defined the size of the saccade as the size of the initial, primary saccade. Either the eye movements with long glissades or drifts were discarded or else the magnitude of the dynamic saccade was carefully calculated. For saccades with overshoot, the saccadic magnitude was defined to be the foot-to-peak angle, i.e., the angle between zero velocity at the start of the saccade and zero velocity at the end of the saccade. Less variability was provided by this definition for the size of the saccade than when the size of the saccade was defined as the angle between the two steady state fixation points. Saccades with dynamic overshoot were treated as two saccades: the main saccade and a small return saccade. We then used the ratio of peakVelocity/ Amplitude, which is the main indicator of the Main Sequence as described by Bahill and Stark (1975), for specifically analyzing and comparing the the group data. This permitted to narrow the otherwise relatively high variability of the single dynamic saccade parameters (see Bahill et al. 1981 for an overview of cause of dynamic variance of saccades).

### Appendix 2

**Complete Table 5.** Correlations ( Spearman Rho) TAP and SDMT and EDSS

Parameter	TAP Series 1	TAP Series 2	TAP Series 3	TAP Series 4
FSS	0.389**	0.220*	0. 177	0.351*
MFIS Cog	0.342*	0. 197	0. 155	0. 276
MFIS Physe	0.385**	0. 224	0. 201	0.345*
MFIS PsySoz	0.502**	0.296*	0. 279	0.419**
HA Cog	0.346*	0. 205	0. 140	0.337*
HA Fat	0.304*	0. 162	0. 118	0.290*
BDI Affect	0. 185	0. 55	0. 092	0. 127
BDI	0.406**	0. 211	0. 188	0.343*
SDMT	0.381*	0.357*	0. 230	0.431*
EDSS	0.326*	0.293*	0.289*	0.357*
9-HPT	0.375*	0.418**	0.466**	0.380*

Note. *significance <0.05, ** significance <0.01
